# Oral administration of Korean propolis extract ameliorates DSS-induced colitis in BALB/c mice

**DOI:** 10.7150/ijms.44834

**Published:** 2020-07-25

**Authors:** Soonjae Hwang, Samnoh Hwang, Minjeong Jo, Chang Gun Lee, Ki-Jong Rhee

**Affiliations:** Department of Biomedical Laboratory Science, College of Health Sciences, Yonsei University MIRAE Campus, Wonju, Gangwon-do 26493, Republic of Korea.

**Keywords:** dextran sulfate sodium, inflammatory bowel disease, colitis, propolis

## Abstract

Inflammatory bowel disease (IBD) is a chronic disorder of the gastrointestinal tract characterized by inflammation. Although IBD is usually treated with anti-inflammatory agents, most of these treatments have limited efficacy. Propolis is a viscous mixture that honeybees produce by mixing saliva and honeycomb with exudate gathered from tree buds, sap flows, or other botanical sources. Although propolis has proved to ameliorate several inflammatory disorders, its therapeutic properties vary by geographical location, plant resources, bee species, and the solvents used in the extraction. In this study, we investigated the effects of Korean propolis in BALB/c mice with dextran sulfate sodium (DSS)-induced colitis. Korean propolis extract was diluted in drinking water, and the BALB/c mice were given DSS for 7 days and Korean propolis for 17 days. The mice were sacrificed on day 17. In the DSS-induced colitis model, Korean propolis significantly decreased the severity of colitis, as assessed by body weight, spleen weight, and colonic length. Furthermore, Korean propolis induced the reduction of the inflammatory cytokine KC, infiltration of immune cells, and colonic hyperplasia in mice with DSS-induced colitis. The Korean propolis also decreased the loss of goblet cells and antibody-reactivity to inflammatory markers in the colons of mice administered DSS. These results demonstrate for the first time that Korean propolis has an ameliorative effect on DSS-induced colonic inflammation in BALB/c mice.

## Introduction

Crohn's disease and ulcerative colitis, collectively known as inflammatory bowel disease (IBD), are chronic inflammatory disorders of the gastrointestinal tract. Both diseases are characterized by chronic epithelial injury and relapsing/remitting inflammation of the intestine 1. The pathogenesis of IBD is not entirely clear, but it has been reported that one pivotal event is a dysregulated immune response against intestinal commensals through a dysfunctional epithelial barrier 2. Although several drugs show clinical efficacy in IBD patients to varying degrees, they are often expensive, exhibit undesired side effects, and become refractory upon continuous usage 3, 4. Natural products are being considered as supplemental therapies to augment existing conventional IBD treatment. These alternative treatments have garnered much interest due to their high accessibility and minimal side effects 5-8.

Orally administered dextran sulfate sodium (DSS) induces colitis in mice and closely mimics human ulcerative colitis pathology 9. DSS induces colitis by damaging the intestinal epithelial monolayer, followed by the dissemination of pro-inflammatory intestinal contents (e.g., bacteria and their products) into underlying tissue, allowing the uncontrolled activation of immune cells 10. Of those cytokines, prominent KC (also referred to as CXCL1) secretion results in the recruitment of neutrophils, which produces further epithelial destruction 11. KC induction is mediated by the NF-κB pathway in several cell types 12-15. DSS also directly induces secretion of IL-8 (also referred to as CXCL8), a functional homologue of murine KC, in a human colonic epithelial cell line *in vitro* 16. It is well known that DSS induces a loss of body weight proportionate to the extent of colonic inflammation 10. Loss of body weight also has a positive correlation with aggravated colitis in IBD patients 17.

Propolis is a wax-like substance gathered by honeybees from the resinous exudates of buds, leaves, branches, and barks found in the vicinity of the beehive 18. Propolis extracts contain numerous compounds known to exert anti-bacterial, anti‐inflammatory, and antioxidant properties 19, 20. Propolis, and therefore propolis extract, contains a mixture of complex pharmacologic compounds that differs greatly depending on the geographical region from which it is collected 19. A report recently showed that the gut microbiome changed differently in rats fed Brazilian propolis and Chinese propolis 21. Therefore, it is important to assess the efficacy of propolis extract from diverse sources to determine whether propolis extracts can be used to reduce intestinal inflammation.

Currently, Brazilian and Chinese propolis extracts have been tested on various inflammation models, including rodent models of colitis. In an acetic acid-induced colitis model, rats given Brazilian propolis extract showed reduced colonic inflammation 22. Similarly, using a DSS model of colitis, Brazilian propolis extract orally administered to Swiss mice decreased colonic inflammation 23. Consistent with the protective effects demonstrated by Brazilian propolis extracts, Chinese propolis extract attenuated high-fat diet-exacerbated colonic inflammation by DSS in rats 24. However, to our knowledge, the effects of Korean propolis have not been studied or examined in the DSS model of colitis in mice. Herein, we show that Korean propolis extract ameliorated DSS-induced colitis in BALB/c mice.

## Results

### Effect of Korean propolis extract on the body weight of DSS-treated mice

**Figure 1:**
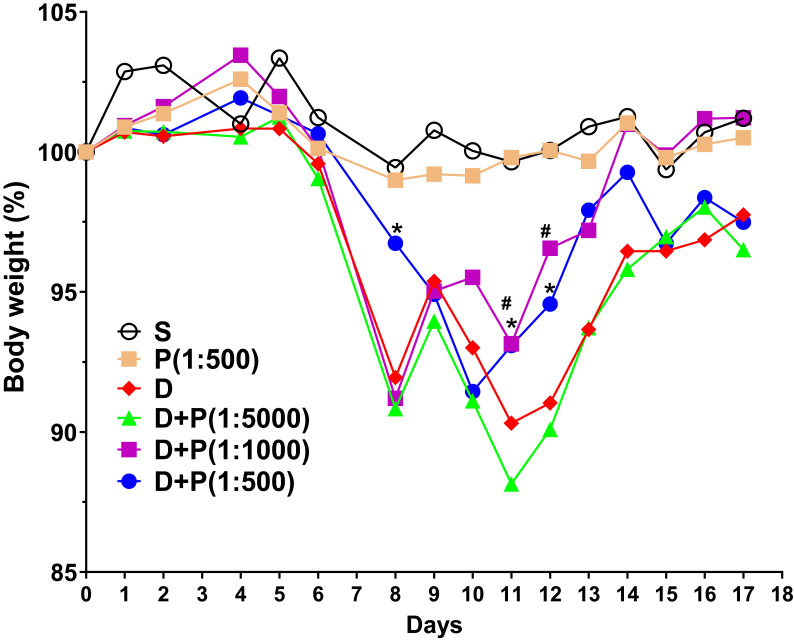
Effect of Korean propolis on body weight changes in DSS-treated mice. The daily body weight of individual mice was normalized to their starting body weights. Sham (S), water alone. P (1:500), Korean propolis extract at 1:500 dilution for 17 days. 2% DSS (D) from day 0-7. D+P, 2% DSS from day 0-7 and dilutions of Korean propolis extract (1:5000, 1:1000, 1:500) from day 0-17. ^#^, a *P* value of < 0.05 for the DSS+propolis (1:1000) group compared with the DSS group. *, a *P* value of < 0.05 for the DSS+propolis (1:500) group compared with the DSS group. n = 10-20 mice per group.

To determine whether Korean propolis extract attenuates DSS-induced colonic inflammation, mice were given 2% DSS in their drinking water for 7 days. Different concentrations (1:500, 1:1000, 1:5000) of Korean propolis extract (referred to hereafter as P500, P1000, and P5000) were also mixed in the drinking water concomitantly for 17 days. Mice given DSS alone for 7 days showed a continuous decrease in body weight until day 11 and then a gradual increase in body weight until day 17 (Figure 1). In contrast, mice given DSS+P500 and DSS+P1000 showed a higher body weight at day 11 and day 12 than mice given DSS alone or DSS+P5000. These results show that DSS-induced loss in body weight recovered more effectively in mice fed propolis extract. Mice given P500 alone showed no difference in body weight compared with sham controls, suggesting that propolis alone at the highest concentration does not overtly affect body weight.

### Effect of Korean propolis extract on water intake, spleen weight, and colon length

**Figure 2:**
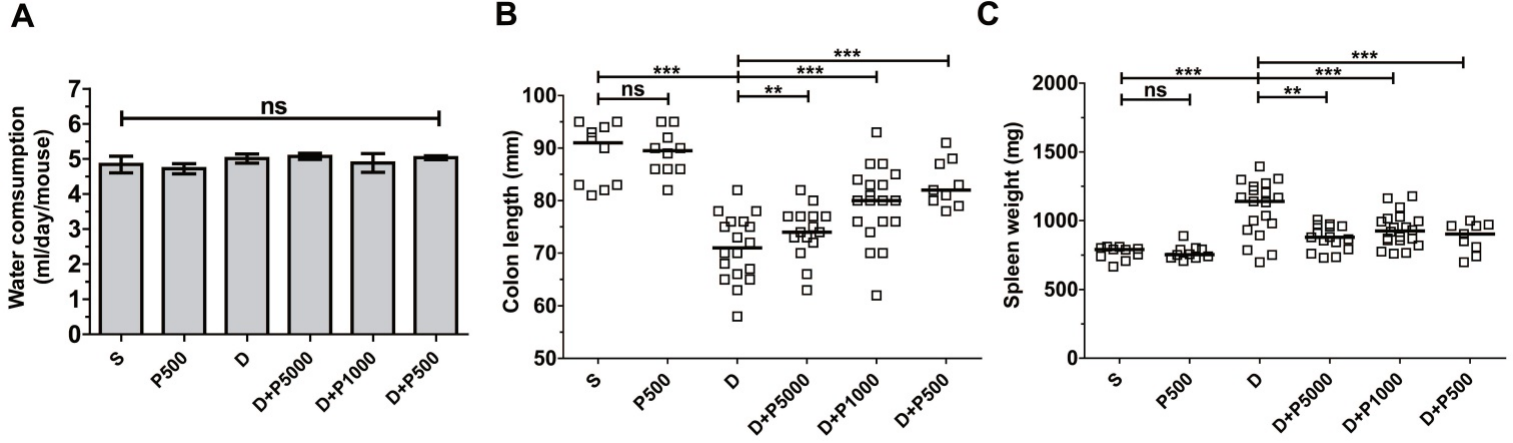
Effects of Korean propolis extract on water intake, colon length, and spleen weight in DSS-treated mice. A, Water consumption (ml/day/mouse). Daily water intake was measured for 17 days. B, Colon length. C, Spleen weight. Each box represents one mouse. Horizontal bar, median. D, DSS; P, Korean propolis extract. S, sham. **P < 0.01, ***P < 0.001. ns, no statistical significance. n = 10-20 mice per group.

Propolis extracts have a distinct odor and taste that could influence water uptake by experimental animals and thus confound the interpretation of the results. Therefore, we assessed the daily water intake by the different groups of mice. The average water consumption during the experimental period of 17 days was 4-5 ml per mouse per day in all groups (Figure 2A), suggesting that propolis consumption was equal among the different experimental groups, suggesting that increased water consumption does not explain the higher recovery after day 11. Mice given Korean propolis extract alone (P500) also showed no difference in water consumption suggesting that, in the current study, propolis did not affect water consumption.

Shortening of the colon length and increased spleen weight are both indirect indicators of DSS-induced colitis severity 25. We assessed both colon length and spleen weight in mice given DSS and/or propolis. We found that on day 17, the colon length was dramatically decreased in the DSS-treated group compared with sham controls (Figure 2B). Mice given DSS+P500 and DSS+P1000, but not DSS+P5000, showed a statistically significant decrease in the shortening of their colons compared with DSS mice. Spleen weight was lower in all groups given both DSS and propolis (DSS+P500, DSS+P1000, and DSS+P5000) compared with mice given DSS alone (Figure 2C). Once again, mice given P500 alone exhibited no difference in either colon length or spleen weight compared with the sham control group. Taken together, the changes in colon length and spleen weight indirectly indicate that Korean propolis extract attenuates DSS-induced colonic inflammation.

### Effect of Korean propolis extract on serum KC levels in DSS-treated mice

**Figure 3:**
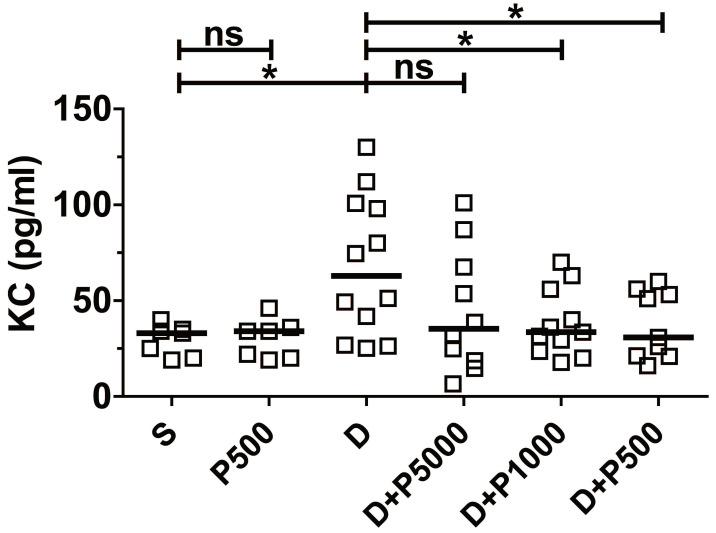
Effects of Korean propolis extract on serum KC levels. Mice were sacrificed on day 17, and sera were isolated through cardiac puncture. Serum KC levels were analyzed using ELISA. Scatter plot. Each box represents one mouse. Horizontal bar, median. *P < 0.05. ns, no statistical significance. n = 7-12 mice per group.

Clinical studies have shown a positive correlation between increased serum levels of the inflammatory cytokine IL-8 and inflammatory disorders 26, 27. Likewise, it was reported that DSS-induced colonic inflammation correlates positively with elevated KC cytokine (functional homologue of human IL-8) in mouse serum 28. Therefore, to investigate whether Korean propolis extract decreases DSS-induced inflammation in the serum, mouse sera were analyzed for mouse KC using ELISA. We found that KC cytokine levels were statistically lower in the DSS+P500 and DSS+P1000 groups than in the DSS group (Figure 3). The DSS+P5000 group also had lower levels than the DSS group, but that difference was not statistically significant. Mice given Korean propolis extract alone showed no difference in serum KC levels compared with the sham control group.

### Effect of Korean propolis extract on colonic inflammation in DSS-treated mice

**Figure 4:**
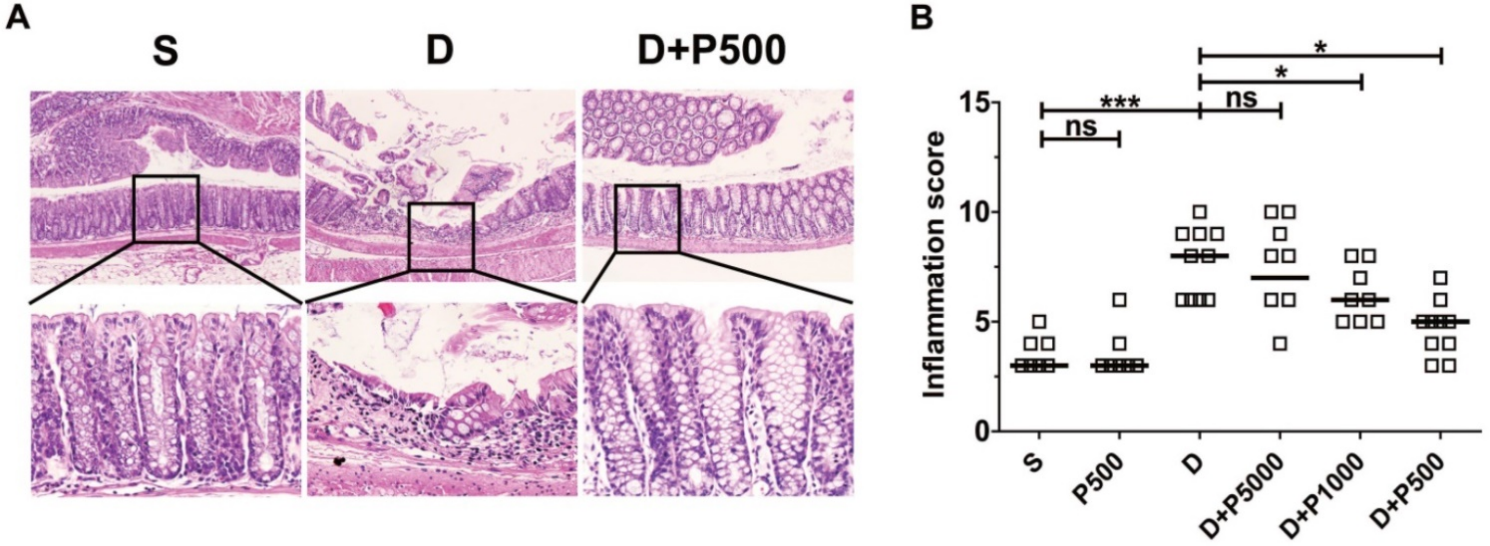
Effects of Korean propolis extract on colonic inflammation in DSS-treated mice. (A) H&E staining of distal colon (large intestine). Magnification x100 (upper panel), x400 (lower panel). Boxed areas were digitally magnified. Results for the propolis (1:500) group are identical to those for the sham group (data not shown). (B) Histologic inflammation scores of the distal colon. Each box represents one mouse. Horizontal bar, median. S, sham; D, DSS; P, Korean propolis extract. *P < 0.05. ***P < 0.001. ns, no statistical significance. n = 7-10 mice per group.

Oral administration of DSS in drinking water induces colonic inflammation 29, 30. To determine if oral administration of Korean propolis extract can decrease DSS-induced colitis, mice were orally administered DSS and Korean propolis extract and colitis was assessed at the end of the experimental period (i.e. day 17). Mice given DSS alone exhibited extensive colonic damage, as determined by H&E staining, whereas mice given DSS+P500 showed colonic damage comparable to that in the sham control (Figure 4A). The colonic inflammation score of the different groups of mice show that the DSS+P500 group had the least damage, and DSS+P1000 also showed protective effects compared with DSS alone (Figure 4B). Mice given DSS+P5000 showed decreased colonic damage, but that difference was not statistically significant. Giving Korean propolis extract alone did not induce colitis in mice. These data indicate that Korean propolis extract ameliorates DSS-induced colitis in a dose-dependent manner.

### Effect of Korean propolis extract on colonic goblet cells

**Figure 5:**
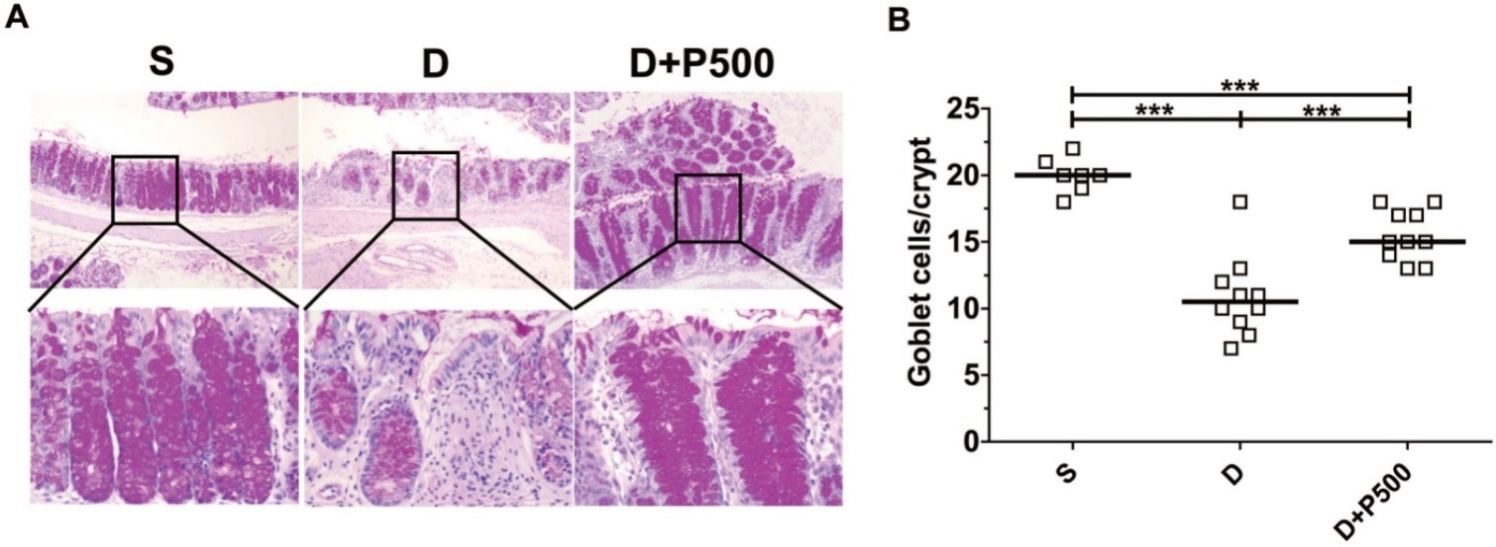
Effects of Korean propolis extract on goblet cell numbers in the distal colons of DSS-treated mice. (A) PAS staining of distal colon (x400). (B) Enumeration of goblet cells per crypt in distal colon. Each box represents one mouse. Horizontal bar, median. S, sham; D, DSS; P, Korean propolis extract. ***P < 0.001. n = 7-10 mice per group.

In the murine DSS colitis model, the severity of colonic inflammation is positively associated with decreased goblet cells within the crypts of the colon 31. Therefore, we assessed the number of goblet cells within the colons of mice treated with DSS and Korean propolis extract using periodic acid-Schiff (PAS) stain, which selectively stains the mucin produced by goblet cells. As expected, mice given DSS alone showed fewer goblet cells than the sham controls, whereas mice given DSS+P500 showed more goblets cells than mice given DSS alone (Figure 5A). Histologic scoring revealed a statistically significant increase in PAS-positive cells per crypt in the DSS+P500 mice compared with the DSS only mice, consistent with the changes observed using H&E staining (Figure 5B). These results indicate that Korean propolis extract can ameliorate goblet cell loss in DSS-treated mice.

### Effects of Korean propolis on the distribution of inflammatory markers in the large intestines of DSS-treated mice

**Figure 6:**
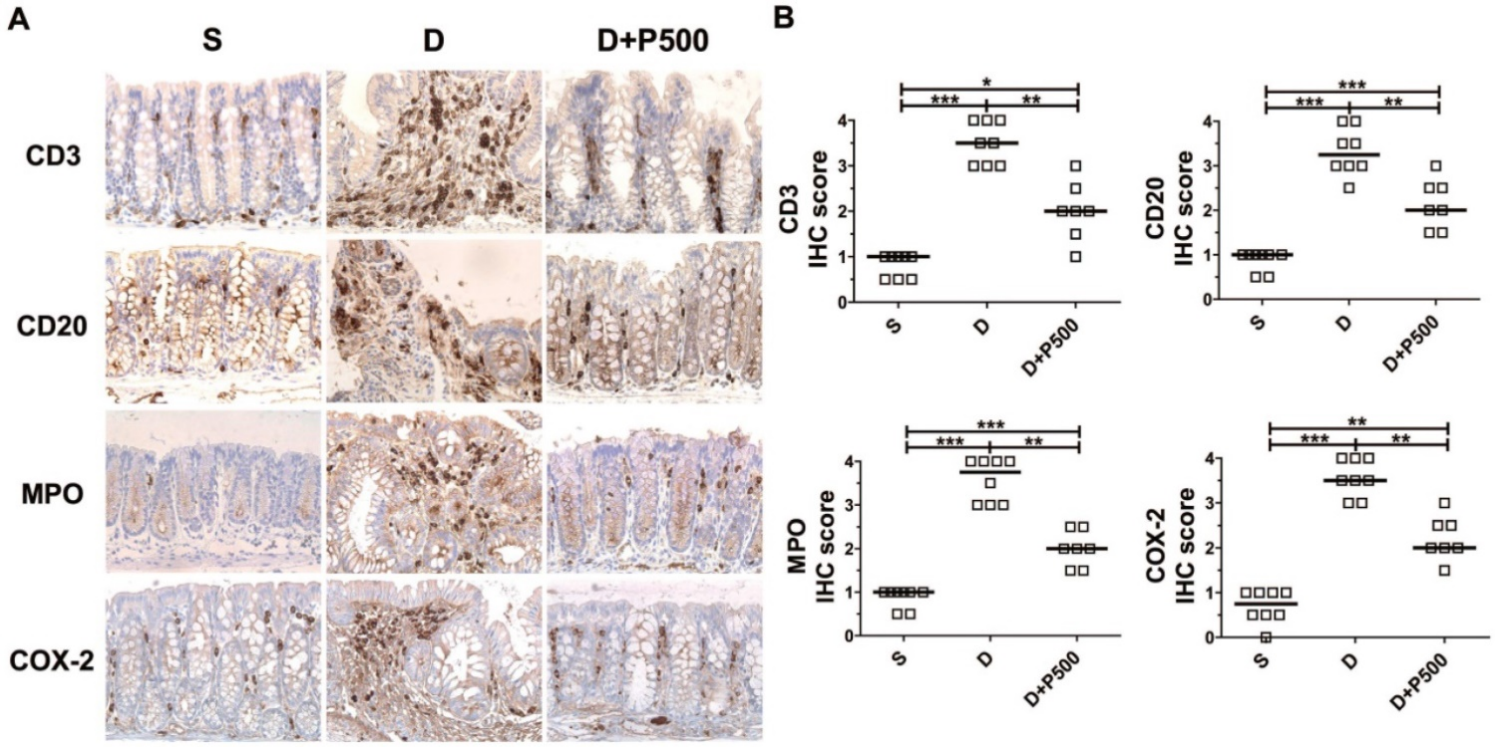
Effects of Korean propolis extract on the distribution of inflammatory markers in the distal colons of DSS-treated mice. (A) IHC staining of the distal colons of mice given DSS and Korean propolis extract. Magnification (X400) (B) IHC scores of distal colon. Each box represents one mouse. Horizontal bar, median. S, sham; D, DSS; P, Korean propolis extract. *P < 0.05, **P < 0.01, ***P < 0.001. n = 7-8 mice per group.

In the DSS-induced colitis model, severe colonic inflammation is positively associated with an increased infiltration of immune cells 32, 33. To determine whether Korean propolis extract can ameliorate immune cell infiltration, distal colon tissues were examined by immunohistochemical staining for the presence of immune cells. We observed that mice treated with DSS alone showed extensive infiltration of T cells (CD3), B cells (CD20), and neutrophils (MPO), as well as increased COX-2 staining indicative of inflammation (Figure 6A). Mice given DSS+P500 showed decreased immune and inflammatory cell infiltration. Scoring the distribution of immune cells and inflammatory cells revealed that the DSS+P500 treated group showed a significant decrease in all four cell types compared with the DSS alone group (Figure 6B). However, the decreased number of immune cells did not reach the levels observed in the sham group, suggesting that even propolis used at the highest dose did not induce complete recovery. The mice given P500 alone showed no significant differences in immune-reactivity for any of the IHC targets compared with the sham group (data not shown). These results suggest that Korean propolis extract ameliorated immune and inflammatory cell infiltration in the colon.

## Discussion

Here we have shown for the first time that a Korean propolis extract can ameliorate DSS-induced colitis in mice. Several recent papers have demonstrated the protective effects of propolis extract from other geographical regions in murine colitis models. Swiss mice given 3% DSS and Brazilian propolis extract showed reduced colonic injury and a reverse in the decreased mucin levels induced by DSS 23. In another study, the protective effects of Brazilian propolis and Chinese propolis were compared in Sprague Dawley rats given 3% DSS 21. Those researchers found that rats receiving orally administrated propolis extract for 1 week prior to 3% DSS treatment showed decreased colitis and reduced inflammatory cytokine (IL-1β, IL-6, and MCP-1) levels. Analysis of the Brazilian propolis and Chinese propolis showed dissimilar chemical compositions, but the protective effect in the DSS model was similar. The same research group also incorporated Chinese propolis into chow, administered it to Sprague Dawley rats with 3% DSS, and found decreased colonic damage 24. Rectal instillation of TNBS (2,4,6-trinitrobenzene sulfonic acid) is another method used to induce colitis in rodents. Using the TNBS model in rats, Goncalves et al. found that Wister rats instilled with 8% (v/v) Brazilian propolis (SLNC 106) showed decreased MPO activity in the colon and decreased colonic inflammation, as well as increased goblet cell numbers 34. Using the acetic acid-induced colitis model, Barbosa Bezerra et al. found that oral administration of Brazilian red propolis reduced colitis in Wistar rats 22. They found significant decreases in MPO concentration, iNOS expression, and tissue damage in the colon. In an earlier study, giving Wistar rats 4% acetic acid and oral propolis also alleviated colitis 35. Taken together, propolis isolated from different geographical regions can dampen colonic inflammation in rodent colitis models irrespective of the differences in chemical composition 21.

Several review articles have highlighted the complex composition of propolis extracts 19, 36, 37. Therefore, it is difficult to determine one particular compound in the Korean propolis that is providing the protection we have documented. We initially focused on caffeic acid phenethyl ester (CAPE), which is a known NF-κB inhibitor derived from a bioactive compound in several types of propolis 38. However, we found that our Korean propolis extract contained approximately 801.1 µg/ml of CAPE, compared with 19,376.8 µg/ml in a commercially available New Zealand propolis extract (unpublished observation). Consistent with this observation, Ahn et al. found that the constituents of propolis collected in various regions of Korea were heterogeneous 39. As is often the case, the beneficial effects of natural compound extracts are likely due to multifactorial components working in synergy and affecting a complex array of inflammatory signaling cascades.

Goblet cells are mucin-secreting intestinal cells that form the mucus layer that protects the gut barrier from extracellular pathogens or chemicals 40. Mucin is an immune barrier in the gastrointestinal tract, and *Muc2*-deficient mice exhibit increased susceptibility to DSS-induced colitis 41. Correspondingly, an *in vivo* study reported that Muc-2 deficiency induced spontaneous inflammation in the colons of mice 41. We found increased numbers of mucin-positive goblet cells in the colonic crypts of mice given DSS and Korean propolis compared with those given only DSS, suggesting that Korean propolis treatment could increase mucin production from goblet cells and thereby confer protection from DSS-induced colonic inflammation. In a recent study, Chinese propolis treatment improved microbial diversity in the gut microbiota of rats given DSS, which was accompanied by the attenuation of DSS-induced colonic inflammation 24. Diet is one of the most pivotal factors affecting the diversity of the gut microbiota 42. Thus, the dysbiosis of gut microbiota caused by DSS might be improved by repeated intake of Korean propolis.

One aspect of the current study that bears attention is the consumption of water during the experimental period. It is well known that rodents prefer certain types of chemicals administered in the drinking water 43, 44. Most animal studies of propolis extract have not consistently documented the uptake of propolis-containing water. In some experiments, mice were fed propolis extract incorporated into their chow, though in many cases, food uptake was not monitored. Similarly, there is a need to monitor DSS consumption, especially when animals are exposed to different therapeutic strategies that could affect consumption 45. In our study, we unequivocally show that water consumption, including water containing DSS and/or propolis, was unaffected.

## Materials and Methods

### Preparation of Korean propolis extract

Raw Korean propolis was collected from a farm in Ulju County, Gyeongsangbuk-do province, Republic of Korea. The geographic coordinates of Ulju County are 35°32'13.99"N (latitude), 129°19'0.01"E (longitude), and 32 ft (elevation above sea level). Raw Korean propolis (200 g) was broken using a grinder and then extracted using 1 liter of 80% (v/v) ethanol for 1 year. During the extraction process, the bottles were manually inverted twice per week and maintained in the dark at room temperature. The extraction mixture was filtered using Whatman No.2 filters (Merck, NJ, USA), and the filtrate stored in polypropylene jars at 4°C until use.

### Mouse experiments

Eight-week-old female BALB/c mice (Raon Bio, Giheung, Republic of Korea) were provided distilled water containing 2% DSS (36-50 kDa, MP Biomedicals, CA, USA) from day 0 to day 7. On day 7, all water bottles were replaced with water containing either Korean propolis extract alone or regular water for the remainder of the study (10 days). The Korean propolis extract was diluted in the drinking water at 1:5000, 1:1000, and 1:500 dilutions and abbreviated as P5000, P1000, and P500, respectively. A total of six groups of mice (10-20 mice per group) were used: sham group (water only from day 0-17), DSS group (DSS from day 0-7, water only from day 8-17) and three DSS+propolis groups (DSS+propolis at P5000, P1000, and P500 from day 0-7, water from day 8-17). The final sixth group was administered P500 alone from day 0-17. Body weights were measured daily. The initial body weight of each individual mouse was set as 100%, and subsequent body weights are expressed relative to that initial body weight. On day 17, all mice were euthanized, and their tissues were harvested for analysis. To determine daily water consumption (ml/day/mouse), groups of mice (5 per group) were given a pre-determined volume of water containing DSS and/or Korean propolis extract, and the remaining water volume measured daily.

### Histology

Mice were euthanized through CO_2_ asphyxiation, and then their colonic tissues were isolated, washed in cold phosphate buffered saline, and fixed in 10% formalin. Thereafter, the tissues were embedded in paraffin and sectioned (5 μm) with a rotary microtome. For histological assessment of colonic damage, formalin-fixed, paraffin-embedded tissue sections were deparaffinized with xylene and stained with hematoxylin and eosin (H&E). Histopathological colitis scores were determined using the parameters in Table 1. To determine goblet cell numbers, formalin-fixed, paraffin-embedded tissue sections were stained using periodic acid-Schiff (PAS) reagent. The number of PAS-positive goblet cells is expressed as number per crypt. Both H&E stained slides and PAS stained slides were evaluated under a light microscope by two independent medical technologists. Slides were photographed by optical microscopy (Leica, Wetzlar, Germany) and rendered using Adobe Photoshop and Leica software.

### Immunohistochemistry

Immunohistochemical (IHC) staining was performed on formalin-fixed, paraffin-embedded tissues. Tissue sections (4 μm) were attached to poly-L-lysine-coated slides, heated, and deparaffinized. Antigen retrieval was performed using sodium citrate (pH 6.0). Slides were immersed in 3% hydrogen peroxide to quench endogenous peroxidase activity and then washed in Tris-buffered saline and Tween 20. Sections were blocked in 5% bovine serum albumin for 1 hour at room temperature and then incubated with primary antibodies to CD3 (clone SP7, Thermo Scientific, USA), CD20 (L26, DAKO, USA), MPO (59A5, Monosan, Netherlands), and COX-2 (CX229, Cayman, USA). Appropriate biotin-labeled secondary antibodies were used to bind the primary antibodies. Bound antibodies were visualized using ABC reagent (Vector Labs, Stuttgart, Germany) and DAB chromophore (Vector Labs, Stuttgart, Germany). Slides were counterstained with hematoxylin. IHC slides were evaluated under a light microscope by two independent medical technologists. IHC scoring for CD3, CD20, MPO, and COX-2 was classified by estimating the percentage of positive cells showing varying staining (from an undetectable level or 0%, to severe staining or >50%), followed by scoring the intensity of staining (0, no staining; 1, weak staining; 2, moderate staining; 3, strong staining, and 4, severe staining). IHC slides were photographed by optical microscopy (Leica, Wetzlar, Germany) and rendered using Adobe Photoshop and Leica software.

### Serum KC quantification

Blood was collected from mice via cardiac puncture, allowed to clot at -4°C, and the sera were stored at -20°C until analysis. Serum levels of KC were quantified using a commercial KC ELISA kit following the manufacturer's instructions (R&D System, MN, USA).

### Statistics

All statistical analyses were performed using the Mann-Whitney test (GraphPad Prism). A value of *P* < 0.05 was regarded as significant.

### Study approval

All animal experiments were approved by the Institutional Animal Care and Use Committee (IACUC) of Yonsei University MIRAE Campus (YWCI-201809-014-01). All experiments conformed to the relevant guidelines and regulations of the IACUC of Yonsei University MIRAE Campus and the Institutional Biosafety Committee of Yonsei University MIRAE Campus.

## Figures and Tables

**Table 1:** Histological parameters for assessment of intestinal inflammation

Feature	Score	Description
Inflammation severity	0	None
	1	Mild
	2	Moderate
	3	Severe
Inflammation extent	0	None
	1	Mucosa
	2	Submucosa
	3	Transmural
Crypt damage	0	None
	1	Base 1/3 damage
	2	Base 2/3 damage
	3	Crypt lost, surface epithelium present
	4	Crypt and surface epithelium lost
